# Measurement of pregnancy rate and foetal loss, and their associations with management practices on reproductive performance of smallholder beef cattle heifers in South Africa

**DOI:** 10.1007/s11250-026-04987-x

**Published:** 2026-03-14

**Authors:** Marble Nkadimeng, Este van Marle-Köster, Nkhanedzeni B. Nengovhela, Fhulufhelo V. Ramukhithi, Johannes M. Rust, Mahlako L. Makgahlela

**Affiliations:** 1 Agricultural Research Council, Germplasm Conservation and Reproductive Biotechnologies, Private Bag X2, Irene, Tshwane, 0062 South Africa; 2 Department of Animal and Wildlife Sciences, University of Pretoria, Hatfield, Pretoria 0002 South Africa; 3 Department of Agriculture, Delpen Building, Corner Annie Botha and Union Street, Riviera, Pretoria 0001 South Africa; 4 Department of Agriculture and Animal Health, University of South Africa, Florida, 1710 South Africa; 5 Döhne Agricultural Development Institute, Stutterheim, 4930 South Africa; 6 Department of Animal, Wildlife and Grassland Sciences, University of the Free State, Bloemfontein, 9301 South Africa; 7 Limpopo Department of Agriculture, Land Reform and Rural Development, Mara Research Station, Polokwane, South Africa

**Keywords:** Calf loss, Herd management tool, Heifer selection, Pregnancy diagnosis, Reproductive management

## Abstract

Understanding reproductive performance of replacement heifers is pivotal for sustainable and profitable farming in any cattle production system. The study assessed reproductive performance of smallholder beef cattle heifers focusing on pregnancy rate (PR); and fetal and calf loss (FCL). Additionally, it investigated herd management factors that influence these traits. A total of 538 heifer records were collected from 40 herds representing five provinces between 2018 and 2019. A secondary application of established seasonal pregnancy diagnosis methods was used to measure PR and FCL. Management factors including body condition score (BCS), age at first mating and calving, breed type, heifer selection, record-keeping, frame size, breeding and calving season were recorded. The Kaplan-Meier method analysed reproductive outcomes, while the Cox proportional hazards model assessed management factors influencing PR and FCL. Results indicated an average of 47% PR and 11% FCL with achievable levels of 60% PR and 0.9% FCL. Majority of heifers conceived between ages four and five years. At three years, 96.19% of heifers had not experienced loss, however, the calf survival probability decreased to 68.17% by five years. The median age for first calving was four, by age five, 72.96% of heifers had calved. The results showed significant effects (*P* < 0.05) for breed type, breeding season, BCS, frame size and heifer selection on PR and FCL. Optimizing PR and FCL in smallholder heifers may depend on improving management practices including selection practices of heifers prior to breeding, maintaining optimum BCS and breeding during favourable environmental conditions.

## Introduction

Reproductive performance of beef cattle heifers is a critical aspect of cattle production as it determines the replacement rate for aging cows and potential genetic progress in the herd (Dickinson et al. 2019). Thus, its success is the core of cow-calf production and its inefficiency limits productivity with herd losses estimated at approximately $43 (R 900) per non-productive heifer at 24 months (Boultona et al. 2017; Moorey and Biase 2020). However, reproductive performance of smallholder beef cattle heifers managed on natural pastures in South Africa (SA) remains unknown, presenting both operational and economic risks to the beef industry (Van der Westhuizen et al. 2020). This gap exists as most existing literature focuses on dairy cattle, while available data on beef cattle primarily originates from controlled research centers (Theron and Mostert 2009; Muller et al. 2018; Grobler et al. 2023).

In heifers, traits such as pregnancy rate, fetal loss or calving rate are among fertility related traits that may be recorded to measure reproductive performance (Dickinson et al. 2019; Copley et al. 2022). Understanding the dynamics of these traits is crucial for finding solutions for continuous improvement of fertility and expanding scientific literature under extensive production including smallholder beef cattle herds (da Silva et al. 2018). Although reproductive performance data from SA smallholder farms is limited, studies in other low-input extensive beef cattle herds provide an understanding of heifer reproductive dynamics in similar systems. Reported findings include 74% pregnancy rate in Brazil (da Silver et al. 2018), an average pregnancy rate of 78% in Mexico and 53% in Zimbabwe (Luna-Nevarez et al. 2014; Washaya et al. 2019). Furthermore, McCosker et al. (2022) reported foetal losses of 7% in Northern Australia and 5% was reported in Brazil (Junior et al. 2019).

The success or failure of reproductive performance in heifers depends on herd management (Crowe et al. 2018). Key management factors such as breeding season, age at first mating and calving, heifer selection and heifer body condition have been known to be associated with herd reproductive performance (Budisatria et al. 2021; Nazhat et al. 2021; Temesgen et al. 2022). These factors directly influence critical traits such as pregnancy rate, pregnancy losses and overall herd productivity (Moorey and Biase 2020). Heifers with adequate fat reserves measured by BCS produce higher leptin levels, which stimulate GnRH release and promote puberty onset (Dickinson et al. 2019). Age at first mating and calving are underlying heifer’s fertility lifespan as they determine the maturity of reproductive systems, which regulates conception success, embryonic loss and calving ease (López-Paredes et al. 2018; D’Occhio et al. 2019). Breeding season optimizes reproductive timing by matching nutrient availability with breeding to support important reproductive activities such as pregnancy success and embryo survival (López-Paredes et al. 2018).

Reproductive success in the beef cattle smallholder system is regarded as a primary limitation. Therefore, understanding herd reproductive performance levels and the management factors influencing performance in beef cattle heifers within the smallholder conditions may contribute knowledge on herd fertility and provide breeding strategies for informed decision-making on replacement heifers and improving smallholder herd productivity. The study assessed the reproductive performance of smallholder beef cattle heifers, focusing on pregnancy rate (PR) and fetal and calf loss (FCL). Additionally, it investigated herd management factors that affect these traits.

## Materials and methods

### Animals and study site

The present study adopted methodological framework established from a broader research investigating reproductive performance of multiparous cows in smallholder herds (Nkadimeng et al. 2022a, b, 2024). The research evaluated smallholder beef cattle farming system and its associated challenges (Nkadimeng et al. 2022a), to define reproductive performance and establish performance benchmarks for multiparous cows within smallholder herds (Nkadimeng et al. 2022b). Moreover, the previous research identified risk factors associated with the defined reproductive performance in smallholder breeding cows (Nkadimeng et al. 2024). In the current study, the investigation is extended to assess reproductive performance of beef cattle heifers in smallholder herds.

Ethical approval was granted by the Ethics Committee (AEC) of the University of Pretoria (NAS339/2020). Data were collected in five provinces of SA (Eastern Cape, Free State, Limpopo, Mpumalanga and Northwest provinces) between 2018 and 2019. The study sites and participating herds were purposively selected based on the provinces’ participation in the High Value Beef Partnerships (HVBP) project (LS-2016–276) and the herds’ availability of handling facilities needed for data collection. A total of 538 heifer records from 40 smallholder beef cattle herds were collected in two seasons: Autumn (March to May) for pregnancy diagnosis and Spring (September to November) for monitoring confirmed pregnancies and recording pregnancy losses (Nkadimeng et al. 2022b, 2024).

### Collection of reproductive measurements

The reproductive parameters for heifer fertility included were PR and FCL. A portable ultrasound scanner [monitor (Ibex pro, EI Medical Imaging, USA; transducer (5 MHz/12 cm depth)] was used to diagnose pregnancy in participating heifers and PR was calculated as the proportion of heifers confirmed pregnant from the total number tested within the herd. Indicator FCL was defined as the percentage of both abortion and calf mortality (Bunter et al. 2013; Nkadimeng et al. 2024). All heifers (*n* = 538) from 40 herds were pooled and analysed for PR. Analysis of the FCL included only a subset of 134 confirmed pregnant heifers followed to term (Table 1). The missing six herds from FCL analysis were either not pregnant or withdrew from the research during follow-ups. Both indicators PR and FCL were obtained at the individual heifer level and later summarized as herd-level averages. For each participating heifer, animal records on age, hip height, BCS, age at first mating, age at first calving, gestation length and breed type were collected. Hip height was measured for each heifer as an indicator of frame size. It was measured by lowering a retractable measuring tape vertically from a fixed point above the squeeze chute and measurements were recorded from this point to the highest part of the heifer’s back located between its hips (McGowan et al. 2014; Nkadimeng et al. 2024). Hip height was categorised as small (< 125 cm), medium (125 to < 140 cm) and large (≥ 140 cm) frame. The BCS was scored using the 1–5 score scale. Age at first mating was the age heifers were first introduced to a bull and age at calving was recorded as the age of heifers successful calving according to the farmer’s records. Gestation length was measured as age in months of the foetus during PD and breed type was recorded according to the specific breed’s physical characteristics and resemblances (Nkadimeng et al. 2022b). All heifers were managed under extensive native pasture grazing.

The study recorded herd management practices including the use of breeding season, knowledge of body condition score prior to breeding, culling of old and unproductive cows, record-keeping, selection of heifers prior breeding and bull-to-cow ratio through guided questionnaire interviews with each farmer. The breeding season was based on farmer’s reported practices with observed variations from continuous breeding, September-February, December-March, August-October, November-February and September-December. The bull-to-cow ratio was categorized as follows: 1 = Ideal (1:30), 2 = Under (1:15) and 3 = Over (1:70). The bull-to-cow ratio categories were based on farmers’ reports on the number of breeding bulls or how they allocate bulls to cows during the mating period. The factors, knowledge of body condition score prior to breeding, culling of old and unproductive cows, selection of heifers prior breeding and record-keeping practices were recorded as binary variables (Yes/No). Heifer selection prior to breeding was recorded based on farmers’ self-reported practices without predefined selection criteria. For farmers who indicated ‘Yes,’ follow-up questions were asked to determine the specific selection factors considered.

**Table 1:** Summary of data collected

Traits	No. Herds	Categories	No. animals	Totals
PR	40	Pregnant	283	538
		Not pregnant	255	
FCL	34	Loss	16	134
		Calved	118	
Questionnaire	40	Farmers	40	40

Total = sum of categories per trait

### Statistical analysis

Data analysis were performed using statistical analysis system (SAS) version 9.4 with summary statistics generated through frequency tables. The quartile range (25th, 50th (median) and 75th) were chosen as summary statistics for determining targeted achievable levels of performance. The indicator FCL was determined by higher value of the first quartile (25%), while the third quartile (75%) was a target for achievable level in PR. The Kaplan-Meier method was applied to analyse the time required for a heifer to achieve specific reproductive outcome (PR and FCL) and accounted for heifers where the events of interest (PR or FCL) did not occur as censored (Andrade 2023). For estimating the time to pregnancy, non-pregnant heifers were treated as censored with time variable defined as the age at first mating. When FCL was the event of interest, the time variable to the event was defined as the age at first mating and heifers that successfully calved were considered censored.

In addition to Kaplan-Meier survival analysis, the Cox proportional hazards regression model (Andrade 2023) was employed to assess the influence of animal (age at first mating and calving, BCS at mating and calving, hip height) and management (insemination month, calving month, frame size, heifer selection, breed, bull-to-cow ratio, and BCS prior to breeding) factors on time-to PR and FCL outcomes. The hazard ratios estimated from the Cox model indicate the relative risk of an event based on the covariates. Each covariate’s hazard ratio (HR) indicates the likelihood of an event (i.e. PR and FCL) occurring. The Cox regression model was defined as:

$$h\left(t\right)=ho\left(t\right).exp({\beta}_{1}{X}_{1}+{\beta}_{2}{X}_{2}+\cdots{\beta}_{k}{X}_{k})$$

$h\left(t\right)$ represents the hazard function at time $t$, while $ho\left(t\right)$ denotes the baseline hazard function which is the hazard when all covariates are equal to zero. The coefficients ${\beta}_{1}+{\beta}_{2}+\cdots{\beta}_{k}$ correspond to the covariates ${X}_{1}+{X}_{2}+\cdots{X}_{k}$ where ${X}_{1}+{X}_{2}+\cdots{X}_{k}$ represent the animal and management factors.

The study additionally included time to first calving in the survival analysis to provide a broader perspective on heifer reproductive performance. The variable was presented descriptively to highlight calving patterns without interfering with the main fertility indicators (PR and FCL). For this analysis, the time variable was the age at first calving and heifers that did not calve were treated as censored.

## Results

**Fig. 1:**
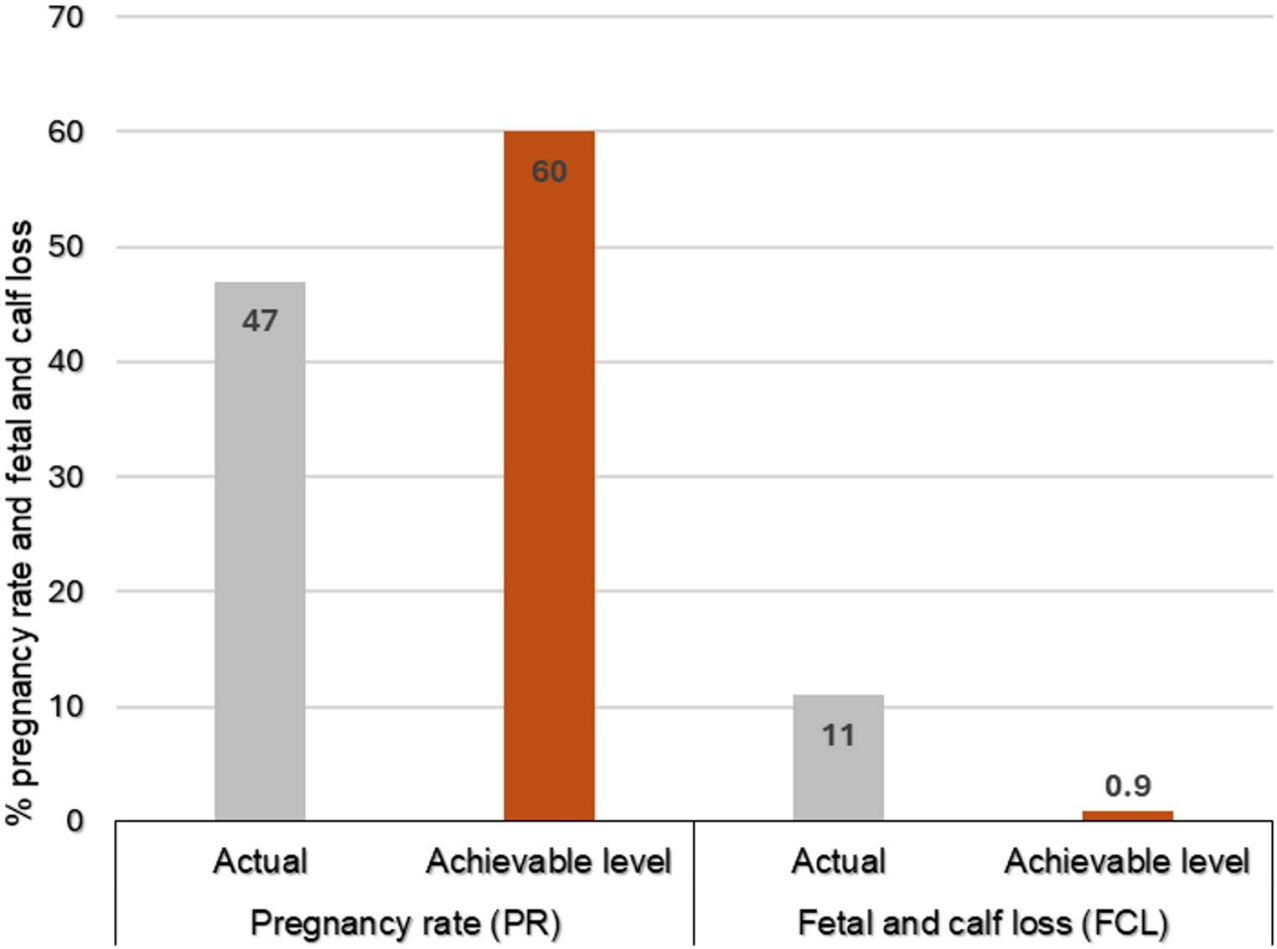
Actual and achievable levels of performance of PR & FCL. The achievable levels were determined by higher value of either the first quartile (25%) or third quartile (75%)

In Table 2 the descriptive statistics of beef cattle heifer parameters in smallholder herds are presented. Results indicate an overall PR of 47.4% with a FCL of 11% across all herds. The achievable levels of performance in these herds were 60% PR and 0.9% FCL (Fig. 1). The majority of heifers at breeding had a BCS of 3 (55.32%), whereas post calving a BCS of ≤ 2 (47.76%) was observed. Moreover, 64.31% of the heifers in smallholder farms are of medium frame and 69.15% were at ages three and four years old at mating. Most herds practice continuous breeding with a bull-to-cow ratio of 1:15 and do not apply heifer selection or pre-breeding BCS.

**Table 2:** Description of beef cattle heifer parameters

Parameters	Categories	% of parameters
**Reproductive measurements**		
Pregnancy rate	Pregnant	47.40
	Not pregnant	52.60
Foetal loss	-	11
**Management factors**		
Age at mating (years)	2	19.70
	3 & 4	69.15
	≥ 5	11.15
BCS at breeding		
	≤ 2	39.59
	3	55.32
	≥ 3	5.95
BCS post calving		
	≤ 2	47.76
	3	46.27
	≥ 3	5.97
Frame size		
	Small	33.09
	Medium	64.31
	Large	2.60
Heifer selection	Yes	44.80
	No	55.20
BCS Prior breeding	Yes	42.19
	No	57.81
Bull to cow ratio	1	27.70
	2	70.45
	3	1.86
Breeding season	Continuous	42.57
	December-February	26.77
	December-March	6.32
	January-March	0.74
	November-February	15.06
	October-March	5.95
	September-December	1.86
	August-October	0.74

% of parameters =frequency percentage of herd parameters

Table 3 shows the Kaplan-Meier survival time to PR and FCL for smallholder beef cattle heifers. The majority of heifers conceived between the ages of four and five years. At age three, 35.04% of heifers were pregnant and by age five 75.07% have successfully conceived. The proportion of heifers not pregnant declined steadily with age, with majority at age two years and decreasing by age five. The survival analysis for the time to FCL in beef heifers indicates that at three years of age 96.19% of heifers had not experienced FCL. However, this survival probability decreased to 89.9% by age four and further dropped to 68.17% by five years of age.

**Table 3:** Kaplan-Meier survival time to pregnancy in smallholder beef cattle heifers

Trait	Survival	Failure	Survival standard error	Survival	Failure	Survival standard error
	PR			FCL		
Age at first mating						
2	0.9052	0.0948	0.0126	-	-	-
3	0.6496	0.3504	0.0216	0.9619	0.0381	0.0187
4	0.4533	0.5467	0.0268	0.8998	0.1002	0.0347
5	0.2493	0.7507	0.0326	0.6817	0.3183	0.0721

Survival probability = the proportion of heifers that have not yet become pregnant or experienced FCL at a given time. Failure probability= represents the cumulative proportion of heifers that have conceived or experienced FCL at that age

The Kaplan-Meier survival analysis (Table 4) for the time to first calving in beef heifers showed that the median age for first calving is four years. By age five, 72.96% of heifers have calved and by age six 93.47% have successfully calved.

**Table 4:** Kaplan-Meier survival analysis for the time to first calving in smallholder beef cattle heifers

Trait	Survival	Failure	Survival standard error
Age at first calving			
3	0.8060	0.1940	0.0342
4	0.4851	0.5149	0.0432
5	0.2704	0.7296	0.0391
6	0.0653	0.9347	0.0235

Survival probability = the proportion of heifers that have not yet calved at a given time. Failure probability= represents the cumulative proportion of heifers that have calved at that age

The cox regression analysis of factors influencing PR measured from age at first mating is shown in Table 5. The Brahman and Beefmaster breed types were projected to have higher likelihoods of becoming pregnant (HR = 4.061; HR = 2.761, respectively). The model predicted an increased PR for heifers that bred between the months of December-March (HR = 17.141), October-March (HR = 13.987) and those bred throughout the year (HR = 13.653). Compared to smaller heifers (HR = 0.3360), heifers with large frame had a higher probability of becoming pregnant (HR = 1.745). Furthermore, the model predicted herds practicing selection of heifers prior breeding to have a higher pregnancy rate (HR = 3.42).

**Table 5:** Cox regression analysis of predictors associated with PR measured from age at first mating

Variable	Cox regression coefficient	SE	HR	*P* value
**Breed**				**0.0267**
Afrikaner Type	0.15208	0.47285	1.164	0.7477
Beefmaster Type	1.01560	0.45167	2.761	0.0245
Bonsmara Type	-0.09843	0.35229	0.906	0.7799
Boran Type	-0.52509	0.40237	0.592	0.1919
Brahman type	1.40139	0.71090	4.061	0.0487
Drakensberg Type	-0.64189	0.46469	0.526	0.1672
Nguni Type	-0.14029	0.36165	0.869	0.6981
Hereford Type	0.90881	0.73790	2.481	0.2181
**Frame size**				**0.0151**
Large	0.55670	0.75578	1.745	0.4614
Medium	-0.0009013	0.47464	0.999	0.9985
Small	0.46831	0.48672	0.3360	1.597
**Bull to cow ratio**	-1.21326	0.28928	0.297	**0.0001**
**Insemination month**				**0.0122**
Continuous	2.61397	1.11494	13.653	0.0191
September-February	2.12838	1.19051	8.401	0.0738
October-March	2.63814	1.20022	13.987	0.0279
August-October	1.94927	1.32158	7.024	0.1402
December-March	2.84146	1.12123	17.141	0.0113
November-February	1.89358	1.22406	6.643	0.1219
September-December	2.36269	1.25919	10.620	0.0606
**Heifer selection**				**0.0010**
Yes vs. No	1.23072	0.37343	3.424	0.0010

Statistically significant at level (< *0.05*). The Cox regression coefficient $\left(\beta\right)$ = logarithm of the hazard ratio, *SE* = Standard Error, *HR* = Hazard ratios represent the relative risk of the event occurring for each category

Table 6 shows the cox regression on the parameters associated with FCL. The results show that heifers with a BCS of 3 at breeding had a significantly lower risk of loss (HR = 0.066) compared to those in BCS 2 (HR = 0.567).

**Table 6:** Cox regression analysis of predictors associated with FCL measured from age at first calving

Variable	Cox regression coefficient	SE	HR	*P* value
**BCS at breeding**				**0.007**
BCS at breeding 2	-0.56805	0.83679	0.567	0.4972
BCS at breeding 3	-2.72567	0.98725	0.066	0.0058
**Reasons for loss**				0.0176
Aborted	19.69118	0.62465	3.562	0.9941
Died/still birth	1.48298	0.62474	1.936	0.8568
**Hip height**				**0.0431**
Large	-0.11018	0.05696	0.896	0.0531
Medium	0.19095	1.05801	1.210	0.8568
Small hip	0.33187	1.16404	1.394	0.7756

Statistically significant at level (< *0.05*). The Cox regression coefficient $\left(\beta\right)$ *SE* = Standard Error, *HR* = Hazard ratios represent the relative risk of the event occurring for each category

In addition, the model predicted (HR = 1.394) higher risk of FCL for heifers with small frame compared to (HR = 0.896) larger frame heifers. Aborted heifers had a higher hazard ratio for FCL (HR = 3.56) compared to death-related loss (HR = 1.93).

## Discussion

The study provided insights into the reproductive performance of smallholder beef cattle heifers as measured by PR and FCL, and further identified factors associated with performance indicators. The findings indicated that management factors including breeding season, BCS, frame size, breed type and heifer selection prior breeding are major contributing factors of the observed 47.76% PR and 11% FCL. Although the industry standard is to breed heifers to calve at two years of age (Day and Nogueira 2013; Dickinson et al. 2018; Damiran et al. 2018), the current study has shown a different trajectory in smallholder beef cattle herds. In these herds, the study revealed majority of the heifer population (75%) to conceive between four and five years of age and only 24% conceived at two years of age.

In other countries such as New Zealand, United States and Ireland the decision to breed heifers past 24 months to calve at 36 months is a management strategy aimed at reducing the risk of calving difficulties, supporting growth of the fetus and ensuring heifers reach their full productive potential (Titterington et al. 2015; Twomey and Cromie 2023). This approach may also be influenced by the observation that progeny from immature dams inseminated at 15 months tend to have a 15–20% lower birth weight (Moorey and Biase 2020; Valiente et al. 2021; López-Valiente et al. 2024). In SA, however, delaying breeding cannot be a management tool as the aforementioned countries achieve pregnancy rates between 78 and 90% during the first estrus cycle, whereas SA reports PR below 50% regardless of the age at first estrus (McFadden et al. 2005; Dickinson et al. 2019; Moorey and Biase 2020).

The concept of reducing the age at breeding must be balanced with production constraints. It is reported that for successful early breeding, heifers should typically achieve at least 60% of their mature body weight to support optimal reproductive performance (Twomey and Cromie 2023). Although recommended, such nutritional management may not always be achievable in smallholder farms, primarily in the tropics (Fordyce and Chandra 2016; Schatz 2012). The expectation of heifers calving at 2 years of age in tropical regions may be constrained by low weight of sexual maturity as a result of fluctuation in nutrition and harsh environment conditions (Copley et al. 2022). While achieving AFC of 24 months may be challenging in tropical regions, the observed median AFC of four years in this study is still higher than in other smallholder farms in similar regions such as in Indonesia (37 months to 38 months) (Budisatria et al. 2019; Warman et al. 2023) and Tanzania (32 months) (Chawala et al. 2017). It is therefore imperative that smallholder farmers invest in management strategies to improve selection for improved performance. One management strategy highlighted by Dickson (2018) and Heslin et al. (2020) emphasize that farmers may select heifers born within the first 60 days of calving as they will be bred with increased morphological and physiological maturity compared to their contemporaries. This reaffirms the importance of selection prior breeding as evidenced by the current findings which highlighted increased pregnancy rate for herds that select heifers prior breeding.

Optimal breeding season has been extensively studied and remains a critical aspect in achieving successful reproductive performance primarily in tropical regions (Dennis et al. 2018; Burrow 2019; Copley et al. 2022). In the present study, breeding between December and March was associated with a higher probability of pregnancy in smallholder beef cattle heifers. Moreover, continuous breeding also resulted in an increased pregnancy rate. The presence of a bull throughout the year may offer advantages as bulls can stimulate regular estrus cycling through pheromonal and physical interactions (Kamal 2023). Continuous breeding season may have beneficial effects, however, a key challenge arises when heifers calve during periods of forage scarcity which is needed to support both their own growth and lactation (Temesgen et al. 2022). Therefore, rather than direct exposure to bulls, farmers may adopt the fence-line contact method proposed by Choudhary et al. (2019) to stimulate estrous activity in heifers from 15-month-old heifers as an alternative approach.

In the current study the model predicted likelihoods of increased PR in Beef master and Brahman breed types. In principle, larger calves at weaning are more capable of gaining weight of up to 25% of their mature weight at puberty (Webster 1989). However, as much as the present study showed the probability of high pregnancy in larger breed type, it is also worth noting that larger breeds survive better where environmental conditions and nutrition are not limiting (McIntosh et al. 2023). Evidence can be seen in the report by da Silver et al. (2018) in tropical regions of Brazil where large heifers reduced BCS from 4.08 ± 0.06 to 3.99 ± 0.04 by end of breeding season due to their higher nutritional requirements as compared to the observed gain in small heifers from 3.71 ± 0.05 to 3.88 ± 0.04. The concept of either keeping larger, medium or small breeds is an important production indicator and may be dependent on the affordability and environment conditions of farmers. The identified BCS 2 as a major factor contributing to FCL in the current study emphasized the importance of adequate nutritional management. The results are in agreement with the report by Zobela, et al. (2011) which documented a 14.91% increase in pregnancy loss among heifers with a BCS of 2. This is likely due to the fact that low BCS at breeding is associated with low secretion of progesterone which is responsible for maintenance of pregnancy till term (Middleton et al. 2019).

Initial investment of a cow begins when the heifer it birthed successfully conceives, which makes early reproductive maturity a critical factor in herd productivity. The current study has highlighted critical flaws in the reproductive performance of replacements heifers’ leading to delayed herd turnover for smallholder beef cattle. The findings provided baseline evidence for extension services, advisory programs and policymakers. It demonstrated that prolonged anestrus and low PR and FCL in smallholder heifers are largely attributable to inadequate nutritional management, suboptimal breeding season and management selection strategies that fail to emphasize early reproductive efficiency. This may delay growth in smallholder beef cattle production system. However, the established 60% PR and 0.9% FCL achievable levels in the current study suggest that there is scope for improvement on reproductive performance of replacement heifers in smallholder farms through better management practices.

## Conclusion

The current study demonstrated key management factors including breeding months, breed type, BCS, hip height and heifer selection to define the recorded PR and FCL in smallholder beef cattle heifers. Notably, majority of the heifers that conceived were between four and five years of age and only 50% calved by the age of four. By six years, 90% of heifers have experienced their first calving. These findings highlighted critical areas for improvement in smallholder beef cattle reproduction management practices. It is recommended that future studies should focus on developing trait guided selection criteria for heifers prior breeding to reduce age at first conception and consider bull-related factors and their influence on reproductive outcomes. Additionally, optimizing nutritional management to ensure targeted mating weight through aligning breeding seasons with favourable conditions could enhance reproductive performance and overall herd productivity in the smallholder beef production system.

## Data Availability

All data analysed during this study are included in the article.
